# Homœopathic League Tracts No. 18

**Published:** 1888-06

**Authors:** 


### Homœopathic League Tracts. No. 18.

Continuing their work of enlightening the public upon Homœopathy, the League publishes this essay on "Allopathic Misconceptions of Homœopathy."

